# Macrophage Recognition of Crystals and Nanoparticles

**DOI:** 10.3389/fimmu.2018.00103

**Published:** 2018-01-29

**Authors:** Masafumi Nakayama

**Affiliations:** ^1^Frontier Research Institute for Interdisciplinary Sciences, Tohoku University, Sendai, Japan; ^2^PRESTO, Japan Science and Technology Agency, Kawaguchi, Japan

**Keywords:** macrophages, phagocytosis, crystals, nanoparticles, inflammation, scavenger receptors

## Abstract

Inhalation of exogenous crystals such as silica, asbestos, and carbon nanotubes can cause lung fibrosis and cancer. Endogenous crystals such as monosodium urate, cholesterol, and hydroxyapatite are associated with pathogenesis of gout, atherosclerosis, and osteoarthritis, respectively. These crystal-associated-inflammatory diseases are triggered by the macrophage NLRP3 inflammasome activation and cell death. Therefore, it is important to understand how macrophages recognize crystals. However, it is unlikely that macrophages have evolutionally acquired receptors specific for crystals or recently emerged nanoparticles. Several recent studies have reported that some crystal particles are negatively charged and are recognized by scavenger receptor family members in a charge-dependent manner. Alternatively, a model for receptor-independent phagocytosis of crystals has also been proposed. This review focuses on the mechanisms by which macrophages recognize crystals and nanoparticles.

## Introduction

Phagocytosis of crystals such as silica, asbestos, monosodium urate (MSU), and hydroxyapatite by macrophages was initially observed by electron microscopy about 40 years ago (1–4). These early studies showed that upon phagocytosis, crystals are not digested but instead cause lysosomal damage. Although the underlying mechanism was unclear, this process was referred to as “frustrated phagocytosis” and was implicated in the pathogenesis of inflammatory diseases such as fibrosis and cancer (5).

**Figure 1:**
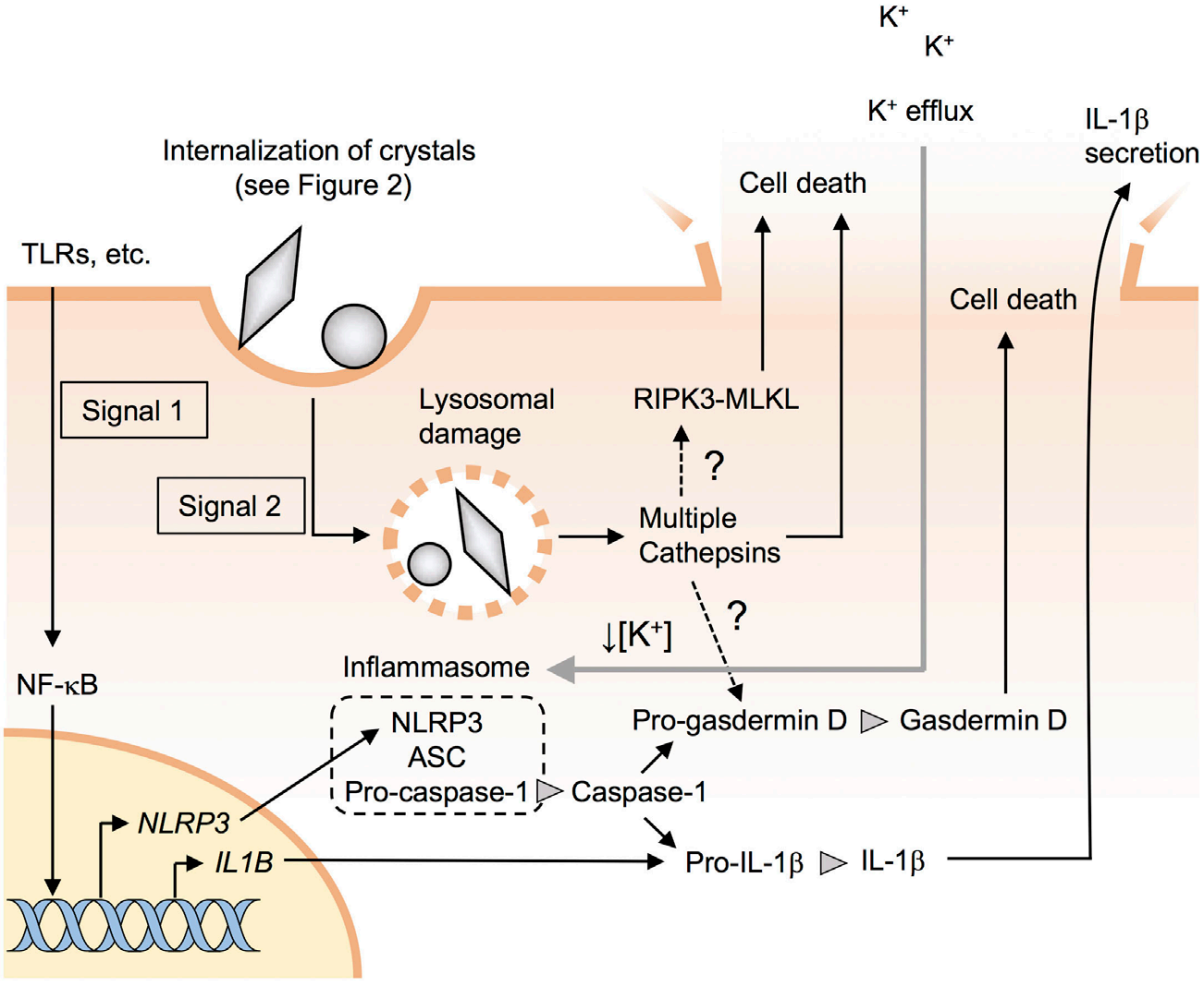
Particle-induced NLRP3 inflammasome activation and cell death. Signal 1 induces pro-IL-1β along with NLRP3 through the nuclear factor-kappa B (NF-κB) pathway. Signal 2 causes lysosomal damages and stimulates the assembly of a complex of multiple proteins including NLRP3, ASC, and procaspase-1, resulting in the formation of inflammasomes. Active caspase-1 processes pro-IL-1β and pro-gasdermin D to mature IL-1β and gasdermin D. Lysosomal damage results in the release of the lysosomal enzyme cathepsins, which may induce NLRP3 inflammasome-independent pyroptotic cell death. Receptor-interacting serine/threonine kinase-mixed-lineage kinase domain-like protein (RIPK3-MLKL) pathway is involved in crystal-induced necroptosis in epithelial cells but not in macrophages.

Recent studies have revealed that silica and asbestos induce IL-1β secretion *via* NLRP3 inflammasome activation in macrophages (6–8). Likewise, various crystals such as MSU, hydroxyapatite, cholesterol, and alum crystals, and nanomaterials such as TiO_2_ nanoparticles and carbon nanotubes (CNTs) have also been reported to induce NLRP3 inflammasome activation in macrophages (7, 9–12). The molecular mechanism for inflammasome activation has been extensively studied and is well summarized in several recent reviews (6, 13–15). Briefly, at least two signals are required for the activation of NLRP3 inflammasome. The first signal (signal 1) is mediated *via* pathogen-associated molecular patterns, damage-associated molecular patterns (DAMPs), or cytokines that trigger nuclear factor-κB (NF-κB)-mediated upregulation of NLRP3 along with pro-IL-1β (Figure 1). The second signal (signal 2) stimulates the assembly of a complex of multiple proteins including NLRP3, ASC, and pro-caspase-1, resulting in the activation of caspase-1. Subsequently, active caspase-1 processes pro-IL-1β to mature IL-1β, which is then released into the extracellular environment through damaged membranes of dying macrophages (Figure 1).

Upon recognition of crystals, macrophage surface receptors transmit signal 1 and/or 2. It is also proposed that the receptor-independent recognition of crystals transmits signal 2. This review summarizes and discusses the recent findings regarding the recognition of crystals and nanoparticles by macrophages.

## Macrophage Surface Receptors

Macrophages express a wide variety of cell-surface receptors in order to recognize and internalize pathogenic particles such as bacteria and apoptotic cells (16–20). For instance, class A scavenger receptors such as SR-A1 (also known as MSR) and MARCO (proposed to be renamed as SR-A6) and class B scavenger receptors such as SR-B1 and CD36 (proposed to be renamed as SR-B2) bind to various polyanionic particles such as bacteria and apoptotic cells (18, 21). Fc receptors such as FcγRIII and complement receptors (CRs) such as CR3 internalize IgG- and complement-opsonized particles, respectively (22, 23), while C-type lectins such as Dectin-1 (also called Clec7a), Mincle (also called Clec4e), and MICL (also called Clec12a) recognize fungi-, mycobacteria-, or dying host cell-associated molecules (17, 24).

Given that it is unlikely that macrophages have acquired the specific ability to recognize crystals and recently emerged nanoparticles through evolution, the abovementioned receptors and opsonins may be responsible for the recognition of such inorganic particles. A common feature of organic particles such as bacteria and apoptotic cells and inorganic particles such as MSU, silica, and titanium is that they have negatively charged surfaces (25, 26), which could be favored by class A and class B scavenger receptors. By contrast, while organic particles harbor various ligands (protein, lipid, etc.) on their surface, the surface of a crystal is remarkably uniform. Therefore, an alternative model for receptor-independent phagocytosis of crystals has also been proposed (27). The mechanisms underlying the recognition of each particle by macrophages are discussed below.

## Recognition of Exogenous Crystals and Nanomaterials by Macrophages

### Silica (SiO_2_) and Titanium (TiO_2_) Particles

Silica, which comprises about 60% of the Earth’s crust, is a major component of sand and rocks and thus is contained in dust and air pollutants (28, 29). Therefore, it can be assumed that most organisms are exposed to crystalline silica (30, 31), and prolonged inhalation of large amounts of crystalline silica dust is known to cause lung fibrosis and cancer (8).

Compared with crystalline silica, amorphous silica is biocompatible and is contained in various foods and medicines (32). However, recent studies have shown that nanoparticles (diameter <100 nm), but not micro-sized particles, can trigger inflammation (32–34). Silica and titanium nanoparticles are the most frequently used nanomaterials (35), and titanium nanoparticles have also been reported to trigger inflammation (36, 37). Under physiological conditions, nanoparticles tend to aggregate irreversibly, resulting in particles of submicron or micrometer size in order to reduce their high surface energy (26, 38, 39). Nevertheless, their toxicity largely depends on their primary size and not on the secondary aggregate size (34, 40) with smaller particles being more toxic. However, the molecular mechanism underlying size-dependent toxicity remains largely unknown.

**Figure 2:**
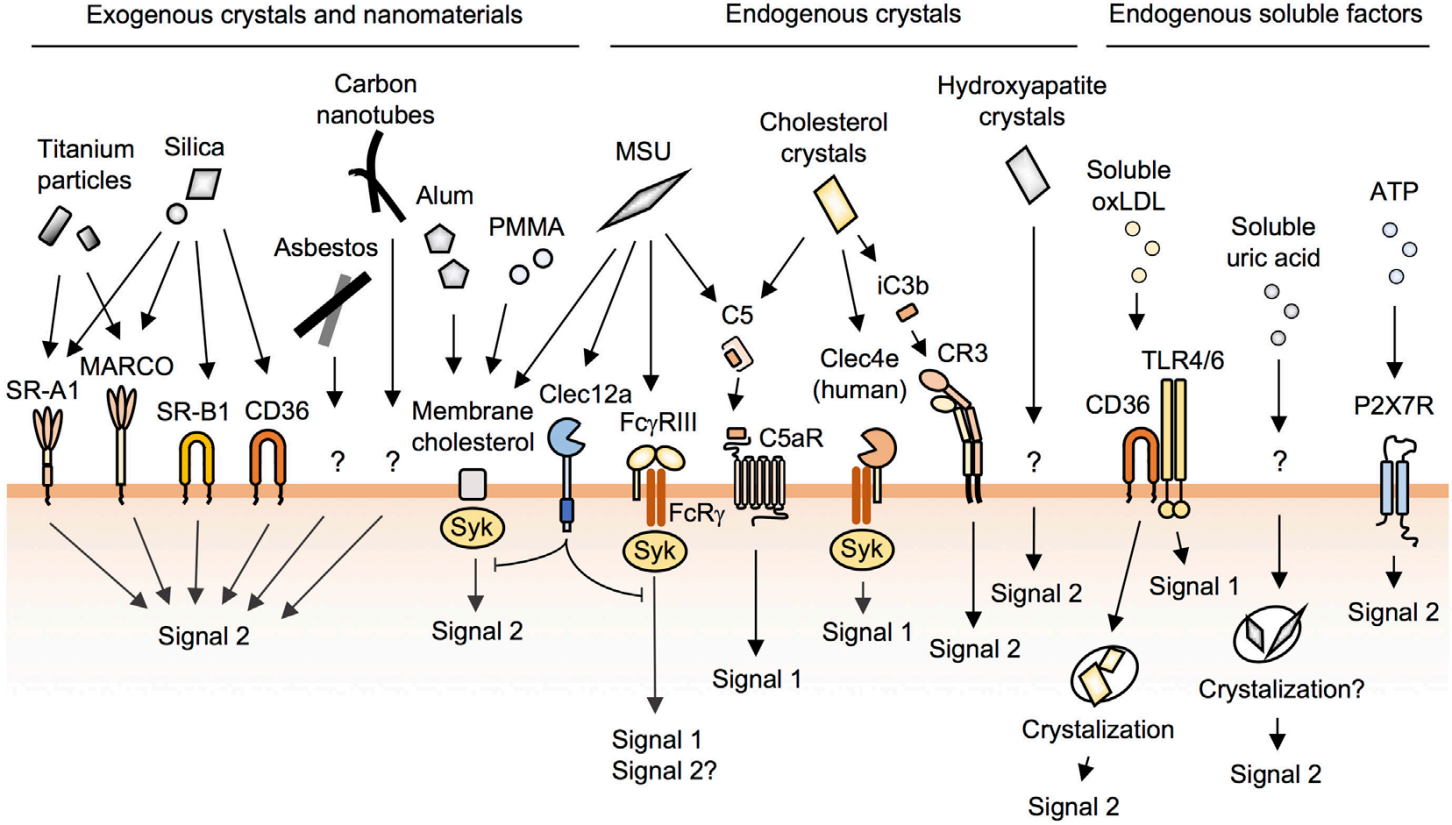
The recognition of crystals and nanoparticles on the macrophage surface. Macrophages recognize and internalize crystals and nanoparticles through cell-surface receptors and membrane cholesterol. Silica particles are recognized by SR-A1, MARCO, SR-B1, and CD36. Alum, poly(methyl methacrylate) (PMMA), and monosodium urate (MSU) crystals bind directly to membrane cholesterol to be internalized. MSU and cholesterol crystals activate complement pathways. Soluble oxidized low-density lipoprotein (oxLDL) is internalized by CD36 and then crystallized in phagosomes. P2X7R does not cause lysosomal damage. In addition to these, many unknown pathways of phagocytosis remain to be identified.

Class A scavenger receptors such as SR-A1 and MARCO are known to bind to silica and titanium particles (Figure 2) (41); however, given that SR-A1- and MARCO-deficient mice and macrophages still show inflammatory responses to these particles (42–44), it seems likely that additional receptor(s) may be involved. Using unbiased functional screening, our laboratory recently identified the class B scavenger receptor member 1 (SR-B1) as a novel silica receptor (Figure 2) (26). In contrast to SR-A1 and MARCO, which bind to both silica and titanium particles (41), SR-B1 binds to silica but not to titanium particles (26). Moreover, SR-B1-deficient macrophages showed impaired internalization of silica and subsequent inflammasome activation (26). However, since SR-B1 binds to both crystalline and amorphous silica irrespective of particle size (26), the recognition by SR-B1 does not account for the size-dependent toxicity of silica particles. On the other hand, Nishijima et al. have recently shown that anti-SR-A1 mAb inhibits THP-1 cell-inflammatory responses to 50-nm silica particles, but not to other sizes of silica particles, suggesting that the recognition by SR-A1 may account for the size-dependent toxicity of silica (45).

The class B scavenger receptor CD36 also binds to silica particles (Figure 2) (26); however, CD36 is not involved in silica-induced acute lung inflammation in mice (26, 46). This is probably due to the marginal expression of CD36 on resident alveolar macrophages (26, 47). Nevertheless, since CD36 is expressed on inflammatory macrophages infiltrating alveolar spaces (47), CD36 may contribute to the chronic lung inflammation.

Because scavenger receptors have only a short cytoplasmic tail (18), they probably work as tethering receptors rather than as signaling receptors following particle recognition. Therefore, co-receptors may also be required in order to internalize particles. Indeed, the ectopic expression of SR-A1 or SR-B1 on non-phagocytic cells enables these cells to bind, but not internalize, to silica particles (26). It has been reported that a chemical inhibitor of Mer receptor tyrosine kinase (MerTK) inhibits IL-1β secretion from THP-1 cells, which use SR-A1 to recognize silica particles. This suggests that MerTK works as a co-receptor of the scavenger receptor (45). Therefore, it would be intriguing to address whether the co-expression of MerTK and the scavenger receptor impart cells with the ability to internalize silica particles.

### Asbestos and CNTs

It is well known that the prolonged inhalation of large amounts of asbestos causes mesothelioma and lung cancer (48). Like silica, asbestos is also efficiently internalized by macrophages, resulting in NLRP3 inflammasome activation and cell death (49). Since asbestos is a silicate mineral, asbestos may also bind to scavenger receptors. Murthy et al. reported that MARCO-deficient mice show less fibrosis following exposure to chrysotile asbestos (50). Although the authors did not show the direct binding of MARCO to asbestos, these data suggest that MARCO may contribute to asbestos-induced lung fibrosis.

Carbon nanotubes are a highly representative product of nanotechnology (51), and although the worldwide production of CNTs is less than that of silica and titanium nanoparticles (35, 51), the production of CNTs has been increasing drastically year after year as they are applied to a wide variety of commercial products including rechargeable batteries and automotive parts (51). Electron microscopy reveals that some CNTs have a needle-like structure reminiscent of asbestos (52, 53). Indeed, recent animal studies have shown that these CNTs have asbestos-like pathogenic behavior (9, 54). For instance, a seminal study by Poland et al. showed that the intraperitoneal injection with multi-walled CNTs (MWCNTs) as well as asbestos causes massive granulomatous inflammation in the diaphragms of wild-type mice (55). Furthermore, Takagi et al. showed that the intraperitoneal injection of MWCNT caused mesothelioma in p53^+/–^ mice (56), while Palomaki et al. found that CNTs and asbestos induce NLPR3 inflammasome activation in human macrophages (57). However, it remains unknown how macrophages recognize CNTs on their cell surface.

### Alum Crystals

Alums (aluminum adjuvants) are widely used for vaccination in both humans and animals (58). It has been reported that upon being internalized, alum activates NLRP3 inflammasomes, which are essential for alum-induced acute inflammation (59, 60). However, recent studies have proposed that the adjuvant effect of alum is mediated *via* NLRP3-independent phagocyte cell death (Figure 1) (61, 62). This occurs when dying cells release their intracellular contents, some of which trigger innate immune responses. Specifically, alum-induced Th2 responses have been reported to be mediated *via* host DNA and uric acid (61, 62). On the other hand, it has been reported that NLRP3 is expressed in the nuclei of Th2 cells and works as a transcriptional regulator of Th2 differentiation (63). Thus, the requirement of NLRP3 for Th2 responses remains controversial.

Shi and colleagues have reported that the phagocytosis of alum as well as MSU crystals (discussed below) is not mediated by cell-surface receptors (64, 65). By using atomic force microscopy, they showed that alum directly binds to membrane lipids, and this lipid ligation activates Syk and PI3K (Figure 2) (64, 65).

In addition to the direct recognition of crystals and nanoparticles by macrophage receptors and membrane cholesterol, these particles may also be *opsonized* and recognized indirectly by macrophages. Indeed, nanoparticles are absorbed by various plasma proteins called the protein corona (66–68). For instance, it has been reported that albumin and complement bind to silica nanoparticles (69), although it remains unknown whether the protein corona contributes to phagocytosis. Some *in vitro* studies have shown that the protein corona does not enhance but rather suppresses the phagocytosis of nanoparticles by macrophages (70, 71). As discussed below, endogenous crystals such as MSU and cholesterol activate complement pathways.

## Recognition of Endogenous Crystals by Macrophages

### MSU Crystals

Dying cells release uric acid, and these crystals trigger inflammation (72). In addition, the saturation of uric acid in body fluids results in the formation of MSU crystals, which trigger macrophage NLRP3 inflammasome activation and are associated with the pathogenesis of gout (10, 73). Early studies reported that MSU crystals activate complement pathways (74, 75), and this has been confirmed by a recent study which showed that MSU and cholesterol crystals (discussed below), but not silica or alum, activate complement pathways (76). Although MSU-activated C5a binds to C5aR, a G protein-coupled receptor (Figure 2), to activate signal 1 (Figure 1) in human monocytes (76), C5aR was not found to contribute to the phagocytosis of MSU (76).

FcγRIII (CD16) has been reported to bind to MSU directly, resulting in the activation of the Syk pathway in human neutrophils (Figure 2) (77). FcγRIII associates with the FcRγ chain, and this receptor complex is a well-characterized phagocytic receptor for IgG-opsonized particles (22); however, the internalization of MSU by FcγRIII has not been demonstrated. Although we and others have observed that MSU crystals have negatively charged surfaces (26, 27), we failed to observe the binding of MSU crystals to scavenger receptors, which can bind to polyanionic particles (26). These results suggest that scavenger receptors may recognize not only surface charges but also shapes and/or substances of particles. Phagocytic receptors for MSU crystals remain to be identified.

Shi and colleagues proposed that MSU crystals bind directly to plasma membrane cholesterols, a driving force for their internalization (Figure 2) (65). This group also proposed that the receptor-independent model can be applied for alum (64) and biomaterial microspheres of poly(methyl methacrylate) (Figure 2) (78). While this is an attractive model for understanding the recognition of crystals and nanoparticles with uniform surfaces by phagocytes, it remains unknown why these particles preferentially bind to phagocytes when cholesterol is present in the membrane of all cell types.

Clec12a (also called MICL, DCAL2, and CLL-1), a C-type lectin receptor, has been recently reported to recognize MSU, but not other particles such as polystyrene, silica, or zymosan (Figure 2) (79). Clec12a has an ITIM in its cytoplasmic domain, and the activation of this receptor has been shown to inhibit Syk signaling. Moreover, Clec12a-deficient mice showed enhanced inflammation in response to MSU (79), although it remains unknown whether Clec12a suppresses phagocytosis of MSU.

A recent study has shown that soluble uric acid also triggers NLRP3 inflammasome activation, although the authors do not exclude the possibility that this activation could be caused by undetectable microcrystals of uric acid (80). It is also possible that internalized soluble uric acid is crystallized in phagocytes just like soluble oxidized LDL as described below (81) (Figure 2). Either way, this finding may propose that uric acid released from dying cells or hyperuricemia directly causes inflammation without crystal deposition.

### Cholesterol Crystals

Cholesterol accumulation leads to the formation of crystals, which have been shown to be engulfed by macrophages in atherosclerotic sinus lesions (12), leading to pro-inflammatory responses through NLRP3 inflammasome activation (10, 82). In addition, early studies have shown that cholesterol crystals as well as MSU crystals activate complement pathways (83, 84). Recently, Samstad et al. showed that cholesterol crystals activate the C5a and the C5aR pathways leading to the upregulation of CR3 (CD11b and CD18 complexes) (Figure 2) (85). Mechanistically, C5aR, a G-protein-coupled receptor, activates ERK and NF-κB pathways (signal 1) (86). These pathways lead to the induction of the expression of CR3, a phagocytic receptor for iC3b-tagged particles (19), which contributes to the phagocytosis of cholesterol crystals (signal 2) (85). Indeed, this group showed that the inhibition of C5 or C3 reduces the phagocytosis of cholesterol crystals by human monocytes (85, 87).

The human, but not mouse, C-type lectin Mincle (also called Clec4e) has been shown to have a cholesterol recognition amino acid consensus (CRAC) motif in its extracellular domain (88). Through this CRAC motif, human Mincle binds to cholesterol crystals, resulting in the activation of pro-inflammatory signals *via* the associated FcRγ chain (Figure 2) (88). Although the FcRγ chain is able to mediate a phagocytic signal (22), it remains unknown whether human Mincle is involved in the phagocytosis of cholesterol crystals.

In addition to cholesterol crystals, soluble oxidized low-density lipoprotein (oxLDL) is internalized by macrophages, and the crystals are then nucleated, resulting in the lysosomal disruption and activation of the NLRP3 inflammasome (81, 82). CD36 is a receptor for oxLDL and is essential for both signal 1 (NF-κB activation in conjunction with TLR4 and -6) and signal 2 (internalization of oxLDL), resulting in NLRP3 inflammasome activation (Figures 1 and 2) (81, 89).

### Hydroxyapatite Crystals

Hydroxyapatite, a basic calcium phosphate crystal, is a major component of bones and teeth. The ectopic deposition of these crystals is predominantly observed in osteoarthritis (OA) joints and is implicated in the pathogenesis of OA (10, 90). In addition, synthetic hydroxyapatite crystals are the widely used biomaterials, although it has been shown that these crystals can trigger local inflammation upon being released from implanted prosthetics (91). Recent studies have reported that hydroxyapatite crystals are internalized by macrophages through unknown mechanisms where they trigger NLRP3 inflammasome activation (Figure 2) (92, 93). NLRP3 is essential for crystal-induced IL-1β secretion *in vitro*; however, the requirement for NLRP3 is only partial in mouse models of arthritis (92, 94) as shown in NLRP3-deficient mice where various crystals such as silica and alum still induce macrophage cell death and inflammation (discussed below). Thus, hydroxyapatite crystal-induced arthritis may be mediated *via* NLRP3-independent macrophage death.

## Particle-Induced Cell Death and Diseases

Crystals cause lysosomal damages, resulting in the release of the lysosomal enzyme cathepsins to cytosol (95), which is the upstream of NLRP3 inflammasome activation (Figure 1). Rock and colleagues have recently shown that multiple cathepsins including cathepsins b, l, x, and s contribute to NLRP3- and caspase-1-independent cell death (Figure 1) (96). However, the downstream mechanisms of action of the cathepsins remain unknown. It would be intriguing to address whether cathepsins directly cause membrane damage or activate pore-forming proteins such as gasdermin D (97–99). It has also been reported that receptor-interacting serine/threonine kinase-3 and mixed-lineage kinase domain-like protein-mediated necroptosis pathways (100) are involved in crystal-induced cell death in epithelial cells (101) but not in macrophages (Figure 1) (96, 102).

Dying cells release DAMPs such as uric acid and ATP (72). As mentioned earlier, uric acid induces inflammation (80). ATP, which is released from pannexin-1, binds to P2X7 receptor to induce cell death and NLRP3 inflammasome activation without causing lysosomal damage (Figure 2) (103). Besides DAMPs, dying macrophages release internalized crystals, which could induce cell death of the neighboring macrophages. This sequential cell death may be more crucial than NLRP3 inflammasome activation in the pathogenesis of crystal-induced chronic inflammation and fibrosis such as arthritis (92, 94), silicosis (104), and asbestosis (105) as these diseases develop in NLRP3-deficient mice.

## Conclusion

Crystals such as silica, asbestos, and MSU cause inflammatory diseases through macrophage activation and cell death. As discussed here, macrophages have been found to recognize crystals *via* cell-surface receptors and/or membrane cholesterol, although these pathways account for only a fraction of crystal phagocytosis. Therefore, many unknown pathways of phagocytosis remain to be identified. While phagocytosis and subsequent lysosomal damage appear to be essential for the pathogenesis of particle-induced-inflammatory diseases, it remains unknown how the physicochemical properties (element, size, etc.) of particles impact lysosomal damage. A better understanding of the molecular mechanisms underlying particle-induced inflammation will provide opportunities not only for the development of therapeutic approaches for incurable silicosis and asbestosis but also for the development of safer nanomaterials in the future.

## Author Contributions

The author confirms being the sole contributor of this work and approved it for publication.

## Conflict of Interest Statement

The author declares that the research was conducted in the absence of any commercial or financial relationships that could be construed as a potential conflict of interest.
